# Probing the potential of bioactive compounds of millets as an inhibitor for lifestyle diseases: molecular docking and simulation-based approach

**DOI:** 10.3389/fnut.2023.1228172

**Published:** 2023-09-26

**Authors:** Kajal Nagre, Nirupma Singh, Chandrika Ghoshal, Gitanjali Tandon, Mir Asif Iquebal, Tarsem Nain, Ram Swaroop Bana, Anita Meena

**Affiliations:** ^1^Division of Genetics, ICAR-Indian Agricultural Research Institute, Pusa Campus, New Delhi, India; ^2^Division of Vegetable Science, ICAR-Indian Agricultural Research Institute, Pusa Campus, New Delhi, India; ^3^Centre for Agricultural Bioinformatics, ICAR-Indian Agricultural Statistics Research Institute, Pusa Campus, New Delhi, India; ^4^Department of Genetics, Maharshi Dayanand University, Rohtak, India; ^5^Division of Agronomy, Indian Agricultural Research Institute, Pusa Campus, New Delhi, India; ^6^ICAR-Central Institute for Arid Horticulture, Beechwal, Bikaner, India

**Keywords:** secondary metabolite, millet, molecular docking, MD simulations, drug likeness, admet

## Abstract

Millets are becoming more popular as a healthy substitute for people with lifestyle disorders. They offer dietary fiber, polyphenols, fatty acids, minerals, vitamins, protein, and antioxidants. The nutritional importance of millets leads to the present *in-silico* study of selective bioactive compounds docked against the targets of lifestyle diseases, *viz*., diabetes, hypertension, and atherosclerosis using molecular docking and molecular simulations approach. Pharmacokinetic analysis was also carried out to analyse ADME properties and toxicity analysis, drug-likeliness, and finally target prediction for new targets for uncharacterized compounds or secondary targets for recognized molecules by Swiss Target Prediction was also done. The docking results revealed that the bioactive compound flavan-4-ol, among all the 50 compounds studied, best docked to all the four targets of lifestyle diseases, *viz*., Human dipeptidyl peptidase IV (−5.94 kcal mol^−1^ binding energy), Sodium-glucose cotransporter-2 (−6.49 kcal mol^−1^) diabetes-related enzyme, the Human angiotensin-converting enzyme (−6.31 kcal mol^−1^) which plays a significant role in hypertension, and Proprotein convertase subtilisin kexin type 9 (−4.67 kcal mol^−1^) for atherosclerosis. Molecular dynamics simulation analysis substantiates that the flavan-4-ol forms a better stability complex with all the targets. ADMET profiles further strengthened the candidature of the flavan-4-ol bioactive compound to be considered for trial as an inhibitor of targets DPPIV, SGLT2, PCSK9, and hACE. We suggest that more research be conducted, taking Flavon-4-ol into account where it can be used as standard treatment for lifestyle diseases.

## 1. Introduction

Millions of individuals throughout the world suffer from the chronic condition of obesity and diabetes, both of which have significant social costs due to their high incidence rates. Insulin resistance, elevated levels of oxidative stress, and increased expression of inflammatory markers are all prominent symptoms of the complicated condition known as obesity, which results in increased body fat mass. A metabolic condition known as diabetes mellitus (DM) is characterized by decreased insulin secretion and dysfunction of pancreatic cells. Metabolic disorders such as diabetes mellitus (DM), hypertension, cardiovascular disease, and obesity all result from being overweight (1). Obesity is currently a global problem; it is a condition where having too much body fat raises the likelihood of developing health issues, which increase the chance of developing chronic illnesses including diabetes and heart disease. The prevalence of obesity and diabetes has increased dramatically over the past few decades as a result of the growing consumption of processed junk food. Diabetes, heart disease, stroke, gall bladder disease, fatty liver, rheumatoid arthritis, and joint diseases are only a few of the long-term health concerns associated with obesity. Foods rich in dietary fibers, beneficial bioactive compounds, and complex carbohydrates are in greater demand due to these health issues (2). The high amount of gluten in cereals makes it difficult to generate nutritious foods or nutraceuticals even though research is being done to biofortify wholegrain cereals like wheat and rice with phenolic acids that have antimutagenic, anti-glycemic, and antioxidative effects (3–5) There is an urgent need to locate new sources of nutraceuticals, natural foods, and other dietary supplements considering the growing lifestyle diseases and the expanding public knowledge of health care and nutrition.

Plant-based medicines are created from unprocessed plant extracts that are complex blends of several phytochemicals. These phytochemicals are used to treat both chronic and infectious disorders because of their unique and complicated biological effects. Even though there is a huge variety of bioactive secondary metabolites present in different plant species, only a small portion of them have undergone extensive research and have been shown as significant sources of bioactive substances. Also, the idea of treating diabetes, obesity, and related diseases with natural treatments has not received much attention. There are more than 5,000 naturally occurring flavonoids that have been identified in a variety of plants. Many studies showed the potential health advantages of natural flavonoids in the treatment of diabetes mellitus (DM) and obesity, and they reveal higher bioavailability and activity on numerous molecular targets. Flavonoids are divided into six main subgroups: flavanols (which include quercetin, kaempferol, and myricetin), flavanones (which include eriodictyol, hesperetin, and naringenin), flavonoids (which include daidzein, genistein, and glycitein), flavones (which include apigenin and luteolin), flavan (including cyanidin, peonidin, and petunidin). Flavonoids may be helpful in the treatment, prevention, and mitigation of a variety of viral illnesses as well as degenerative illnesses like cancer, diabetes, obesity, and other age-related illnesses (6, 7). According to accumulated epidemiological data, dietary flavan-3-ols have a significant effect in lowering the risk of Type II Diabetes Mellitus (8, 9).

Millets are the sixth most-grown cereals in the world, including pearl millet, foxtail millet, finger millet, and other minor millets. Due to their distinct characteristics of being a C_4_ plant with high photosynthetic efficiency, high capacity for producing dry matter, and ability to grow under the most challenging agro-climatic conditions where other crops like sorghum and maize fail to yield, millets outperform all other cereals (10). The millets renamed as Nutri cereals contain alkaloids, flavonoids, terpenes, polyphenols, etc., compared to many other kinds of cereal, including barley, rice, maize, and wheat. Because of their distinctive and complex biological effects, bioactive substances are employed to treat both chronic and infectious diseases. The millet-identified bioactive constituents include gallic acid, protocatechuic acid, p-hydroxybenzoic acid, vanillic acid, syringic acid, ferulic acid, trans-coumaric acid, caffeic acid, sinapic acid, quercetin, and proanthocyanidins (condensed tannins) (11, 12). Even though plant species have a wide range of bioactive secondary metabolites, only a small percentage of them have undergone in-depth study. Recent *in-silico* studies have described the promising effects of bioactive compounds of millets in treating metabolic diseases like diabetes and obesity, hypertension, and cardiovascular disease (13–16).

The identification of bioactive compounds is greatly aided by *in silico* investigations, which also provide several benefits, including minimization of time and expense required for this process. It can be costly and time-consuming to synthesize and test several chemicals as is required by traditional experimental procedures. On the other hand, *in silico* techniques make use of computer simulations and computational models to screen and forecast the activity of hundreds or even millions of chemicals, reducing the number of prospective candidates for further experimental validation. By offering information on molecular interactions, lowering expenses, and improving the likelihood of finding possible therapeutic drugs, they supplement experimental approaches. In the present study, four proteins were used as target receptors Human Dipeptidyl Peptidase (DPPIV), Sodium-Glucose Cotransporter-2 (SGLT-2), Human Angiotensin-Converting Enzyme (hACE), and Proprotein Convertase Subtilisin Kexin type 9 (PCSK9). The aim of the study is to explore bioactive compounds with antidiabetic, antihypertension, and antiatherosclerosis properties in millets using *in-silico* approaches. The selected bioactive compounds were analyzed for pharmacokinetics and physiochemical properties, and MD simulations were carried out to evaluate the binding stability, conformation, and interactive ways between the ligands and target protein. Hence, we aim to investigate the pharmacological activities of the bioactive compounds from millets against diabetes mellitus, atherosclerosis, and hypertension through pharmacokinetics and pharmacological properties, and molecular modeling methods.

## 2. Materials and methods

### 2.1. Macromolecule as target preparation

The three-dimensional X-ray crystallographic structures for targets that play an extremely significant role in diabetes mellitus, atherosclerosis, and hypertension were retrieved from The Research Collaboratory for Structural Bioinformatics (RCSB)^1^ and saved in PDB format (Table 1). The water molecules were deleted and polar hydrogen was added with correct partial charges in the target. The four targets chosen were Human Dipeptidyl Peptidase (DPPIV), Sodium-Glucose Cotransporter-2 (SGLT-2) for diabetes, Human Angiotensin-Converting Enzyme (hACE) for hypertension, and Proprotein Convertase Subtilisin Kexin type 9 (PCSK9) for atherosclerosis based on literature search because these proteins play important roles in lifestyle diseases and the search for their inhibitors from millets will help in dietary interventions (13–16).

**Table 1:** Target with their Protein Data bank ID (PDBID) and amino acid chain used for molecular docking.

Sr. No.	PDB ID	Target or Macromolecule	Chain with Amino acid
1	7VSI	Sodium-glucose Cotransporter-2 (SGLT-2)	A chain 672AA
2	1J2E	Human Dipeptidyl Peptidase IV(DPPIV)	A chain 729 AA
3	2PMW	Proprotein convertase subtilisin Kexin type 9 (PCSK9)	A chain 126 AA
4	2XY9	Human Angiotensin-converting enzyme (ACE)	A chain 585 AA

### 2.2. Ligand selection

The bioactive compounds such as carotenoids, alkaloids, flavonoids, coumarin, and phenol were docked for antidiabetic, antihypertension, and antiatherosclerosis properties based on a literature search. Their structures were retrieved from ZINC DATABASE and PubChem in SDF format which were later converted into PDB format using OPEN BABLE. The 2D structure, PubChem ID, and type of bioactive compound details are given in Table 2. The FDA-approved drug empagliflozin was used as an antidiabetic positive control, while ramipril and atorvastatin were used as standards for antihypertension and antiatherosclerosis studies, respectively (17, 18).

**Table 2:** The 50 compounds studied with their 2D structure and PubChem ID.

Sr. No.	Compound	PubChem Id	2D Structure	Type of bioactive compound	Reference
1	Flavan-4-ol	439712	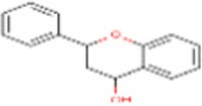	Flavonoid	(11)
2	β-cryptoxanthin	5281235	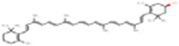	Carotenoid	(12)
3	Daidzein	5281708	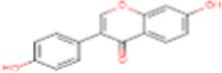	Isoflavone	(19)
4	Naringenin	932	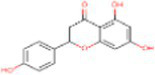	Flavone	(20)
5	Formononetin	5280378	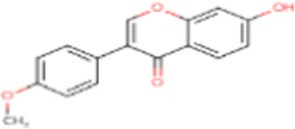	Isoflavone	(19)
6	Violaxanthin	6384269	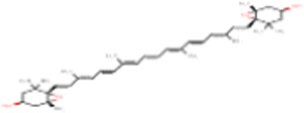	Carotenoid	(20)
7	Zeaxanthin	5280899	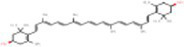	Carotenoid	(11)
8	Quercetin	5280343	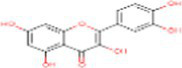	Flavonoid	(20)
9	Luteolin	5280445	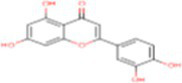	Flavones	(20)
10	Phthalic acid	1017	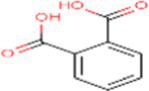	Benzene dicarboxylic acid	(21)
11	Trans- sinapic acid	637775	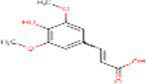	Hydroxycinnamic acids	(22)
12	Dihydroquerectin	471	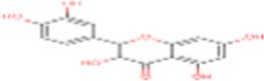	Pentahydroxy flavone	(20)
13	p-coumaric acid	637542	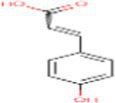	Phenylpropanoid	(20)
14	Caffeic acid	689043	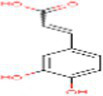	Polyphenols	(22)
15	3,4Dihydroxybenzoic acid	72	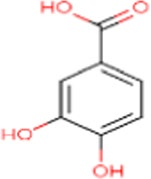	Benzoic acid	(20)
16	Isovitexin	162350	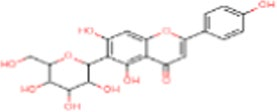	Glycosyl compound	(20)
17	Anthocyanin	56928084	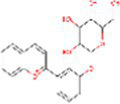	Flavonoids	(20)
18	3, 7 Dimethylquerctin	5280417	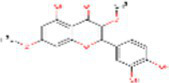	Flavonoids	(23)
19	Apigenin	5280443	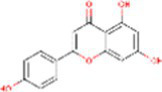	Flavonoids	(9)
20	Tricin	5281702	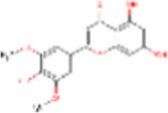	Flavonoids	(20)
21	Isorhamnetin-3-0-glucoside	5318645	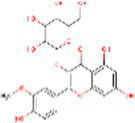	Sesquiterpenoid	(24)
22	Xylotriose	91873341	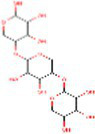	Oligosaccharide	(25)
23	Lucenin-1	44257923	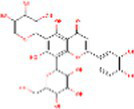	Flavones	(23)
24	Orientin	5281675	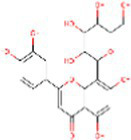	Flavonoids	(19)
25	Myricetin	5281672	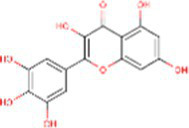	Flavonoids	(22)
26	Vitexin	5280441	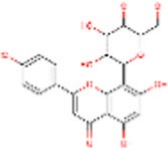	Flavone glycoside	(22)
27	Syringic acid	10742	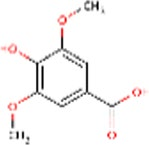	Benzenoids	(20)
28	Hexadecanoic acid	5282743	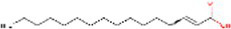	Saturated fatty acids	(21)
29	Chlorogenic acid	1794427	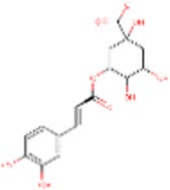	Hydrocinnamoyl derivatives	(20)
30	Xylotetraose	10230811	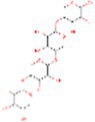	Tetrasaccharide	(25)
31	Gentisic acid	3469	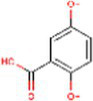	Hydroxybenzoic acid	(22)
32	α tocopherol	86472	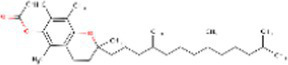	Phenols	(25)
33	Saponarin	441381	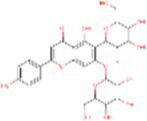	Flavonoid	(19)
34	Xylobiose	439538	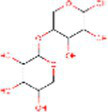	Glycoside hydrolase	(26)
35	Vanillic acid	8468	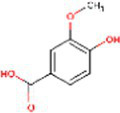	Benzoic acid derivative	(22)
36	Epigallocatechin	72277	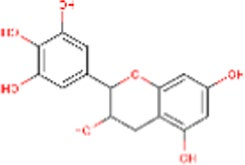	Flavonoid	(22)
37	Catechin	9064	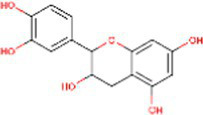	Flavonoid	(19)
38	γ- tocopherol	92729	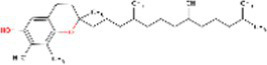	Phenols	(27)
39	Gallic acid	370	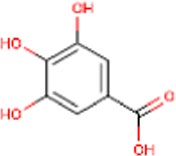	Phenolic acid	(22)
40	Kaempherol	5280863	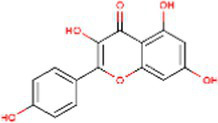	Flavonoids	(20)
41	Epicatechin	107905	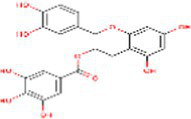	Flavonoid	(22)
42	Procyanidin B1	11250133	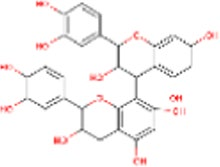	Polyphenols	(22)
43	Cinnamic acid	444539	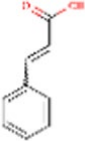	Unsaturated carboxylic acid	(20)
44	Violanthin	442665	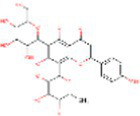	Flavonoid	(22)
45	Proanthocyanidin	107876	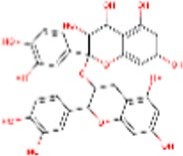	Polyphenol	(22)
46	Ferulic acid	445858	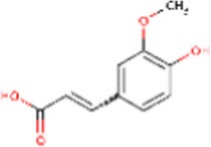	Hydroxycinnamic acids	(20)
47	Atropine	174174	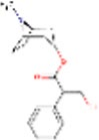	Alkaloid	(26)
48	p-Hydroxybenzoic acid	135	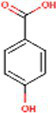	Phenolic acid	(22)
49	Scopolamine	3000322	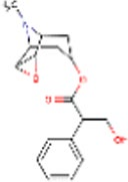	Alkaloid	(26)
50	Proanthocyanidin	107876	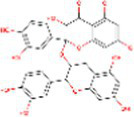	Condensed tannins	(22)

### 2.3. Molecular docking

The process of “molecular docking” explores the potential links between molecules interacting under topographical restrictions or energy considerations to match the two molecules to the optimal interaction conformation (28). Molecular docking is the most modern, efficient, and cost-effective method for creating and testing pharmaceutical compounds (7). Computer-aided tools have become sophisticated drug discovery techniques that can be used to filter drugs from bioactive chemicals found in a variety of therapeutic plants (29). In the present study, in-silico molecular docking is done by using the software Autodock Vina and Autodock Tools. In this study, ligands were kept flexible while proteins were kept rigid. AutoDock was used to create grid boxes to prepare PDBQT files for proteins and ligands, among other intermediary stages. Docking is performed using 10 runs of the Lamarckian Genetic Algorithm. The grid was placed in a box whose coordinates were *x*, *y*, and *z* having 2,048,383 total grid points per map with a spacing of 1.0 Å. The dimensions of the grid box were set as *X* = 126, *Y* = 126, and *Z* = 126, and the center grid box was set with the coordinates as center *x* = 48.648, center *y* = 59.931, and center *z* = 31.936. We used the feature-rich molecular modeling application Discovery Studio Visualizer to view, share, and analyze data. The best docking outcomes were analyzed using Biovia Discovery Studio Visualizer, which is also useful for seeing and assessing predicted protein–ligand interactions.

### 2.4. Molecular dynamics simulations

MD simulation is important to practice the existing drug discovery developments as it assists in a better understanding of molecular structure-to-function relationships (30, 31). In this work, MD simulations have been carried out to evaluate the binding stability, conformation, and interactive ways between the ligands and target protein. This simulation was investigated for receptor-ligand complexes for 100 ns via GROMACS (Groningen Machine for Chemical Simulations) software version 2021 (31, 32). GROMACS is a non-commercial molecular simulation package that is useful in performing simulations of proteins, lipids, and nucleic acids (32). Firstly, the topology of the protein and the ligand was created using CHARMM36 force fields (33). For protein topology, the generation GROMACS program was used, while for ligand topology, CGenFF was used.^2^ CGenFF (CHARMM General Force Field) program performs atom typing and assignment of parameters and charges by analogy in a completely automated fashion (33, 34). The complex was immersed in a dodecahedron box of simple point charge (SPC) water molecules. The solvated system was neutralized by adding counter-ions. Energy minimization of the solvated structures was done using the steepest descent and conjugate gradient algorithm till the maximum force reached below 100 KJ/mol/nm. To equilibrate, the system was then subjected to position–restrained dynamics simulation (NVT and NPT) at 300 K for 100 ns. Finally, this system was subjected to the MD production run for 100 ns at 300 K temperature and 1 bar pressure. For trajectory analysis, various parameters were computed using GROMACS. These included Root Mean Square Deviation (RMSD), Root Mean Square Fluctuation (RMSF), Potential Energy, SASA (solvent accessible surface area), and Molecular mechanics Poisson–Boltzmann surface area (MM/PBSA) calculation.

### 2.5. Pharmacokinetics properties

#### 2.5.1. ADME prediction

Medicinal chemistry and pharmacokinetics of small compounds are demonstrated by ADME. Conducting drug metabolism and pharmacokinetics (DMPK) research, also known as ADME (Absorption, Distribution, Metabolism, and Excretion) investigations, is a crucial step in the discovery and development of new drugs (18). SWISSADME and admetSAR open-source tools are used for ADME analysis (35).

#### 2.5.2. Toxicity analysis

The ProTox-II website studies the toxicity LD_50_ value and toxicity class. The LD_50_ is the fatal dose at which 50% of the tested population is fatal after ingesting the substance. By providing the SMILES from PubChem, the appropriate chemical can be studied in the online database known as pkCSM.^3^ The website offers information such as whether a substance is Ames positive and thus mutagenic. It forecasts whether a specific substance is linked to skin sensitivity or not and suggests whether a particular drug will affect liver functions or not (18).

#### 2.5.3. Drug-likeness properties

The Drug-likeness property is predicted by Lipinski’s Rule. To determine whether a chemical compound has pharmacological or biological activities as an orally active drug in humans, Lipinski’s Rule, a refinement of drug-likeness, is used.

#### 2.5.4. Target prediction

The observed phenotypic effects are the result of the activity of bioactive small molecules, such as metabolites, being modulated by their binding to proteins or other macro-molecular targets. To understand the molecular processes behind the bioactivity of bioactive small compounds and to foretell any adverse effects or cross-reactivity, it is crucial to map their targets. We can computationally find new targets for uncharacterized compounds or secondary targets for recognized molecules. Swiss Target Prediction^4^ is a web service that uses a collection of 2D and 3D similarity measurements with known ligands to precisely predict the targets of bioactive compounds (36).

## 3. Result

### 3.1. Molecular docking

**Figure 1:**
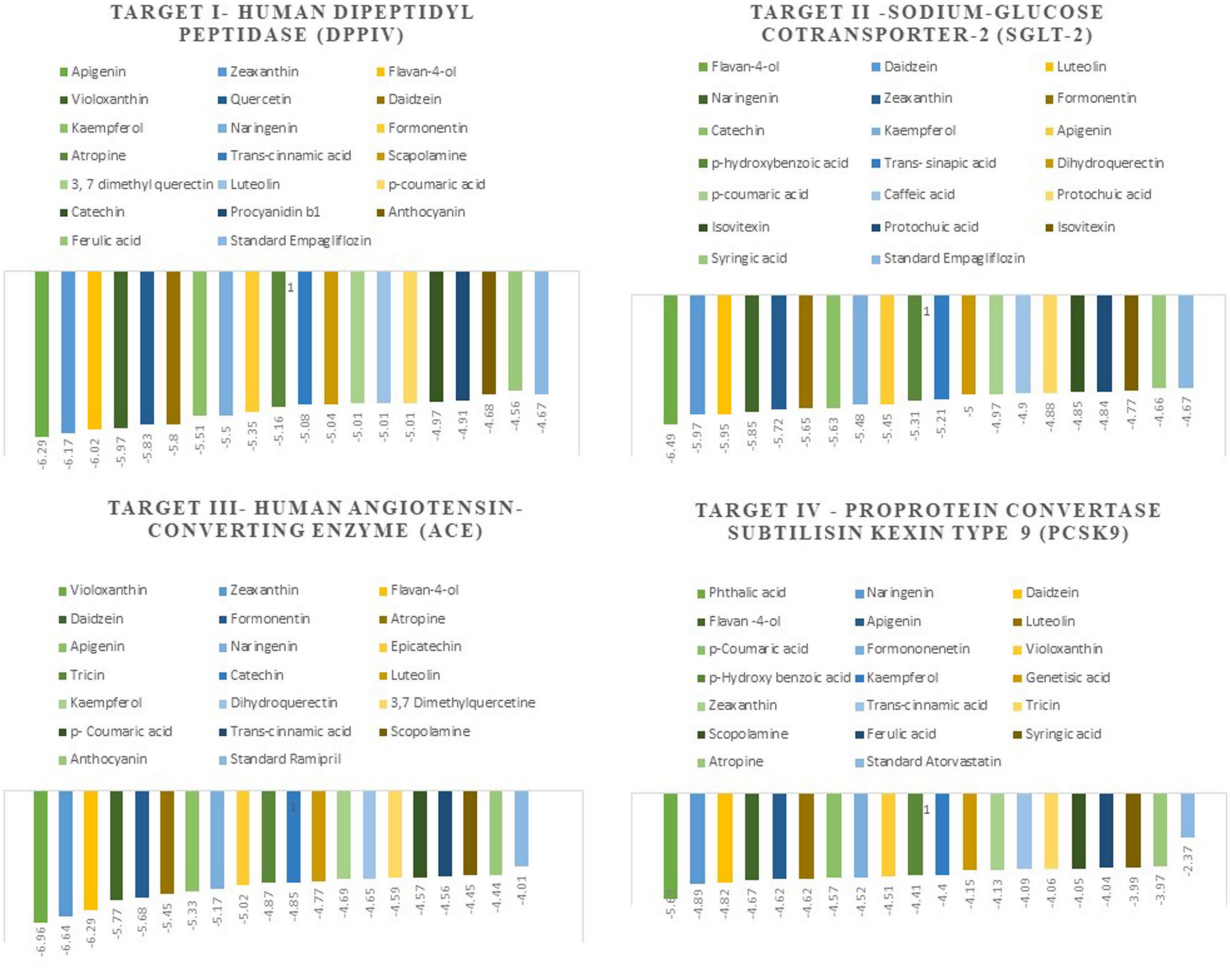
The top 20 compounds docked against the four selected targets Hamann Dipeptidyl Peptidase (DPPIV), Sodium-Glucose Cotransporter-2 (SGLT-2) Human Angiotensin-Converting Enzyme (ACE), and Proprotein Convertase Subtilisin Kexin type 9 (PCSK9) with their binding energies against their respective positive controls.

The docking scores indicate how well the ligands fit into the active site of the target, and the more negative the value, the better the affinity of both ligand and target. The Autodock tools are used for ligand-target interactions and are used to analyze the outcome of docked compounds (37). In our study, 50 bioactive compounds docked against the four targets Human Dipeptidyl Peptidase (DPPIV), Sodium-Glucose Cotransporter-2 (SGLT-2) for diabetes, Human Angiotensin-Converting Enzyme (HACE) for hypertension, and Proprotein Convertase Subtilisin Kexin type 9 (PCSK9) for atherosclerosis. Out of 50 compounds, 20 compounds showed better binding energies with the chosen targets against their standard as shown in Figure 1.

#### 3.1.1. Target I-DPPIV

Molecular docking facilitates the evaluation of the biological effects of small compounds by predicting binding affinity against the target protein. In the current docking studies of 50 bioactive compounds that docked against the DPPIV, the apigenin, zeaxanthin, flavan-4-ol, and violaxanthin showed better binding energy −6.29 kcal mol^−1^, −6.20 kcal mol^−1^, −5.94 kcal mol^−1^, −5.29 kcal mol^−1^, respectively than standard empagliflozin −4.65 kcal mol^−1^. Of the inhibitors studied, the number of hydrogen bonds formed between the target and inhibitor depicts the stable complex formation; apigenin formed four hydrogen bonds with the target, and the rest of the compound formed one hydrogen bond. The amino acid involved in this bond formation were Pro475, Pro510, Lys512, Ile529, Phe559, Arg560, and Asn562, with 24.26 μM inhibition constant. Zeaxanthin hydrogen bond formation involved amino acids Ile102, Val121, Lys122, Phe240, and Ala707, with 28.44 μM inhibition constant, whereas flavan-4-ol formed bond with Leu90, Asn92, Phe95. Ile102, and 44.17 μM inhibition constant. Violaxanthin formed a bond with Val303, Ala465, and Lys466 amino acid residues and 132.40 μM inhibition constant as shown in Table 3.

**Table 3:** Molecular interaction profiling and docking score of the top four bioactive compounds against selected targets in comparison with positive controls.

Sr. No.	Compound	Binding Energy (kcal mol^−1^)	Inhibition Constant (uM)	Hydrogen bond	Amino acid involved
Target I – Human Dipeptidyl Peptidase (DPPIV)					
1	Apigenin	−6.29	24.26	4	Pro475, Pro510, Lys512, Ile529 Phe559, Arg560, Asn562
2	Zeaxanthin	−6.20	28.44	1	Ile102, Val121, Lys122, Phe240, Ala707
3	Flavan-4-ol	−5.94	44.17	1	Leu90, Asn92, Phe95. Ile102
4	Violaxanthin	−5.29	132.40	1	Val303, Ala465, Lys466
5	Standard Empagliflozin	−3.73	1.84	4	Val121, Lys 122, Gln 123, Trp124, Tyr 211, Asp 739
Target II – Sodium-Glucose Cotransporter-2 (SGLT-2)					
1	Flavan-4-ol	−6.49	17.57	2	Asn75, His80, Phe98, Glu99, Val157, Tyr290, Gln457
2	Daidzein	−5.97	41.73	3	Ser156, Val144, Ala446, Ala447, Gln451, Leu452, Tyr455, Phe504
3	Luteolin	−5.95	43.47	3	Asn75, His80, Thr87, Val95, Phe98, Glu99
4	Naringenin	−5.85	51.60	1	Val444, Ala446, Ala447, Gln451, Leu452, Tyr 455, Phe504
5	Standard Empagliflozin	−4.67	379.50	1	Tyr410, Glu421, Val425, Leu428, Trp429, Phe432
Target III – Human Angiotensin Converting Enzyme (hACE)					
1	Violaxanthin	−6.96	7.89	1	Leu194, Pro198, Lys478, Tyr481, Trp486, Pro500, Arg501, Tyr619
2	Zeaxanthin	−6.64	13.65	1	Glu134, Ile138, Leu194, Phe196, Pro198, Lys199, Lys478
3	Flavan-4-ol	−6.31	23.70	3	Trp59, Tyr62, Ala63, Asn66, Ala356, Trp357, Asp358, Tyr360
4	Daidzein	−5.77	5.77	3	Asp121, Glu123, Arg124, Tyr135, Leu139, Ile204, Ala207, Ala208, Ser219, Ser516
5	Standard Ramipril	−4.01	1.15	1	Trp59, Tyr62, Ile88, Trp357, Asp358, Tyr360
Target IV – Proprotein Convertase Subtilisin Kexin type 9 (PCSK9)					
1	Phthalic acid	−5.69	67.61	5	Arg97, Gln101, Arg104, Arg105
2	Naringenin	−4.89	259.51	3	Ala62, Lys 83, Leu 135, Lys 136
3	Daidzein	−4.82	290.97	3	Glu84, Leu 88, Gly11, Pro120
4	Flavan-4-ol	−4.67	378.10	1	Ala62, Leu135, Lys136
5	Standard Atorvastatin	−2.37	18.18	1	Ala62, Arg97, Leu135, Lys136, Pro138

#### 3.1.2. Target II – SGLT-2

All the bioactive compounds that are docked against the macromolecule SGLT-2, from the flavan-4-ol, daidzein, luteolin, and naringenin showed more negative binding energies −6.49 kcal mol^−1^, −5.97 kcal mol^−1^, −5.95 kcal mol^−1^, and − 5.85 kcal mol^−1^, respectively than standard Empagliflozin with −4.67 kcal mol^−1^ value. The more the negative value, the more stability in the complex. The flavan-4-ol formed two hydrogen bonds with the amino acids Asn75, His80, Phe98, Glu99, Val157, Tyr290, and Gln457 with 17.57 μM inhibition constant. Daidzein formed three hydrogen bonds with amino acid Ser156, Val144, Ala446, Ala447, Gln451, Leu452, Tyr455, and Phe504 with 41.73 μM inhibition constant. The luteolin also formed three hydrogen bonds with amino acids Asn75, His80, Thr87, Val95, Phe98, and Glu99 with a 43.47 μM inhibition constant. Naringenin formed one hydrogen bond with Val444, Ala446, Ala447, Gln451, Leu452, Tyr 455, and Phe504 amino acids with 51.60 μM inhibition constant. All the values are depicted in Table 3.

#### 3.1.3. Target III-hACE

In the docking analysis, violaxanthin, zeaxanthin, flavan-4-ol, and daidzein showed the property of hACE inhibitors. The more negative the binding energy value the more stable the complex formed. Compared to conventional Ramipril (−4.01 kcal mol^−1^), the bioactive compound violaxanthin (−6.96 kcal mol^−1^), zeaxanthin (−6.64 kcal mol^−1^), flavan-4-ol (−6.31 kcal mol^−1^), and daidzein (−5.77 kcal mol^−1^) displayed higher binding energies with the target. The violaxanthin interaction with the target formed one hydrogen bond with the involvement of Leu194, Pro198, Lys478, Tyr481, Trp486, Pro500, Arg501, and Tyr619 amino acid residues and 7.89 μM inhibition constant. The zeaxanthin also formed one hydrogen bond with Glu134, Ile138, Leu194, Phe196, Pro198, Lys199, and Lys478 aa residues with 13.65 μM inhibition constant. Flavan-4-ol binds with the Trp59, Tyr62, Ala63, Asn66, Ala356, Trp357, Asp358, and Tyr360 and forms three conventional hydrogen bonds with the target with 23.70 μM inhibition constant. The Daidzein also interacted strongly with the target forming three hydrogen bonds with Asp121, Glu123, Arg124, Tyr135, Leu139, Ile204, Ala207, Ala208, Ser219, and Ser516 amino acid residues having inhibition constant of 5.77 μM as shown in Table 3.

#### 3.1.4. Target IV – PCSK9

In the present study, the inhibitor phthalic acid showed more binding interaction with target PCKS9 than standard Atorvastatin −2.37 kcal mol^−1^ with −5.69 kcal mol^−1^ binding energy. The interaction was made by five hydrogen bonds with amino acid residues Arg97, Gln101, Arg104, and Arg105 with an inhibition constant of 67.61 μM. Whereas, naringenin formed three hydrogen bonds with Ala 62, Lys 83, Leu 135, and Lys 136 amino acid residues and − 4.89 kcal mol^−1^ binding energy, with 259.51 μM inhibition constant. Daidzein binds with the target and formed three hydrogen bonds with Glu84, Leu 88, Gly11, and Pro120 amino acids and had 290.97 μM inhibition constant. The flavan-4-ol with −4.67 kcal mol^−1^ binding energy binds with amino acid residues Ala62, Leu135, and Lys136 forming one hydrogen bond with 378.10 μM inhibition constant, as shown in Table 3.

**Figure 2:**
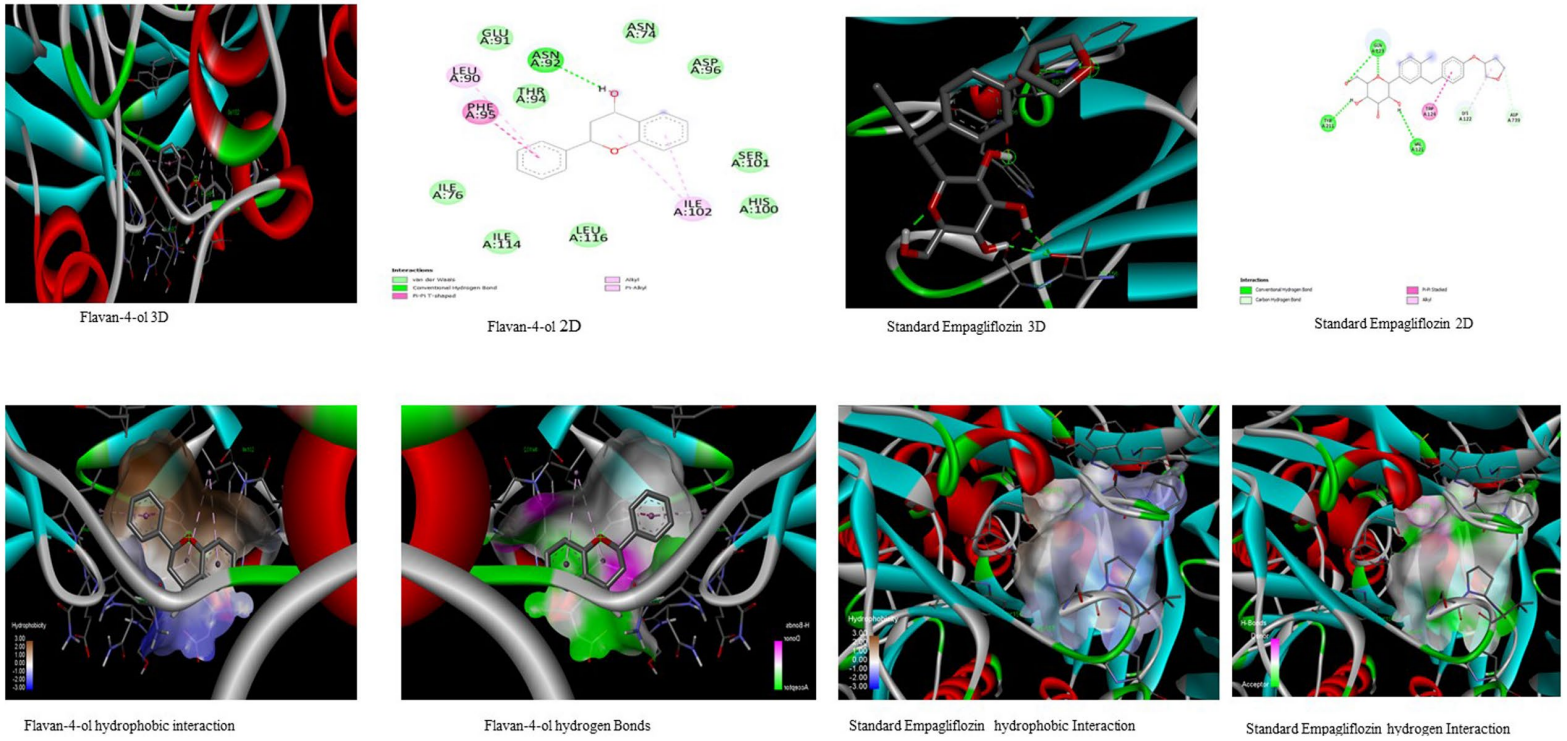
Visualization of binding interaction and hydrogen and hydrophobic bonds formed between the flavan-4-ol with Target I Human Dipeptidyl Peptidase (DPPIV) against their positive controls.

**Figure 3:**
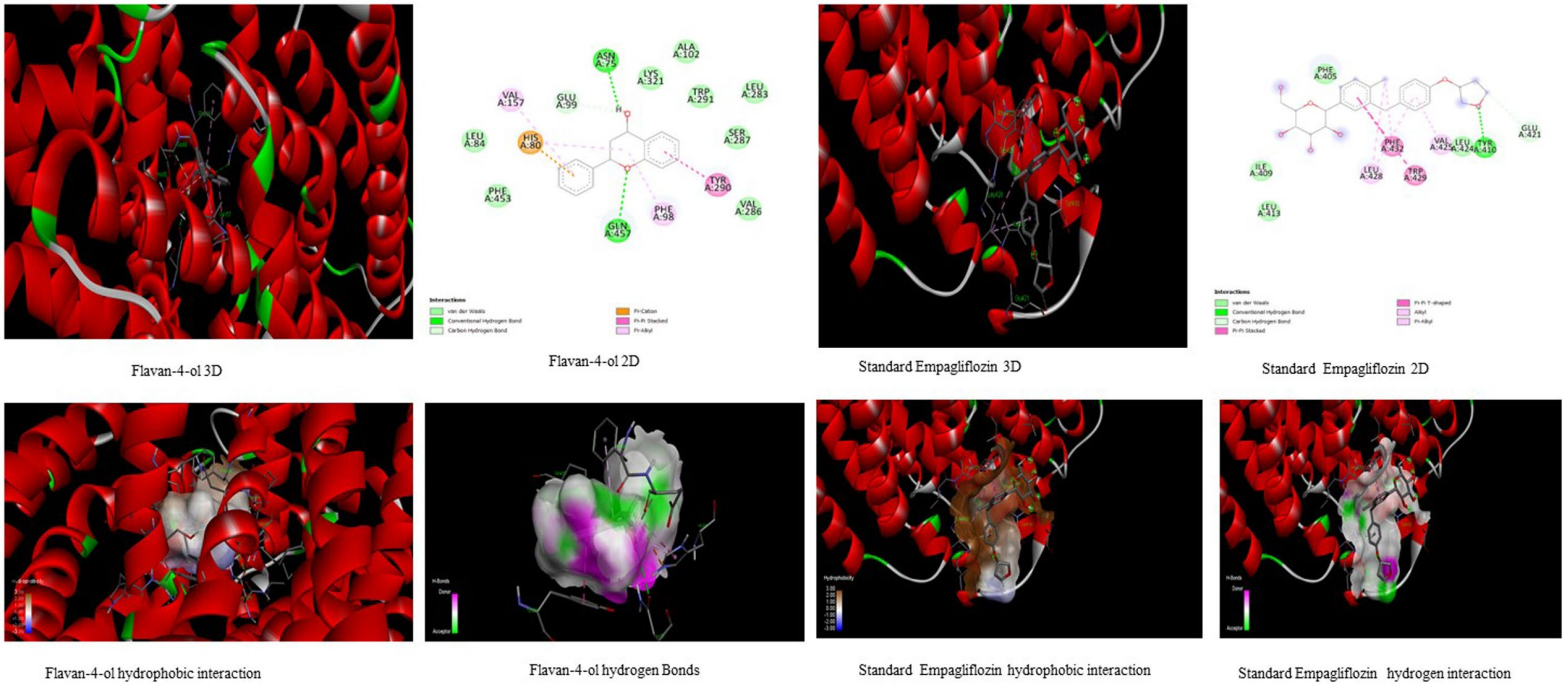
Visualization of binding interaction and hydrogen and hydrophobic bonds formed between the flavan-4-ol with Target II Sodium-Glucose Cotransporter2 (SGLT2) against their positive controls.

**Figure 4:**
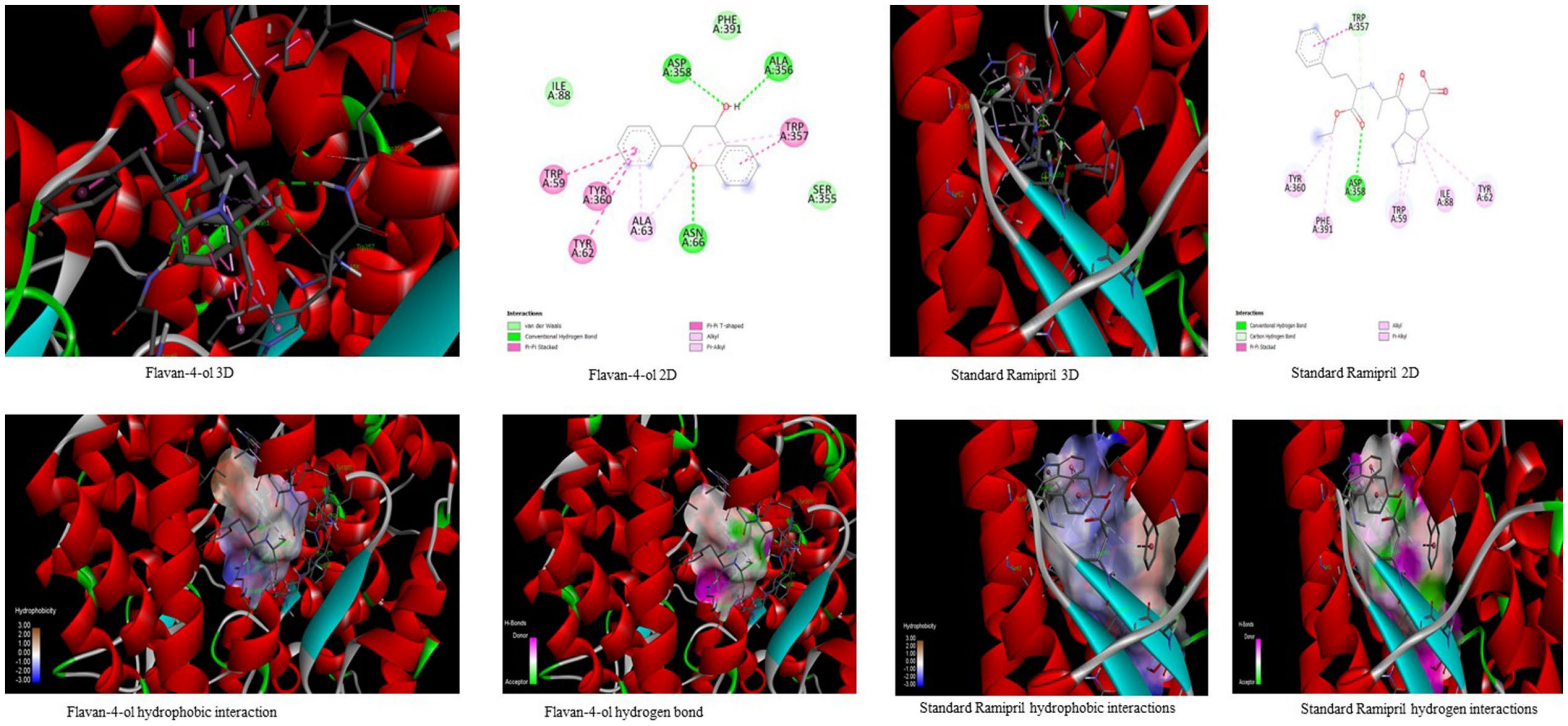
Visualization of binding interaction and hydrogen and hydrophobic bonds formed between the flavan-4-ol with Target III Human Angiotensin-Converting enzyme (ACE) against their positive controls.

**Figure 5:**
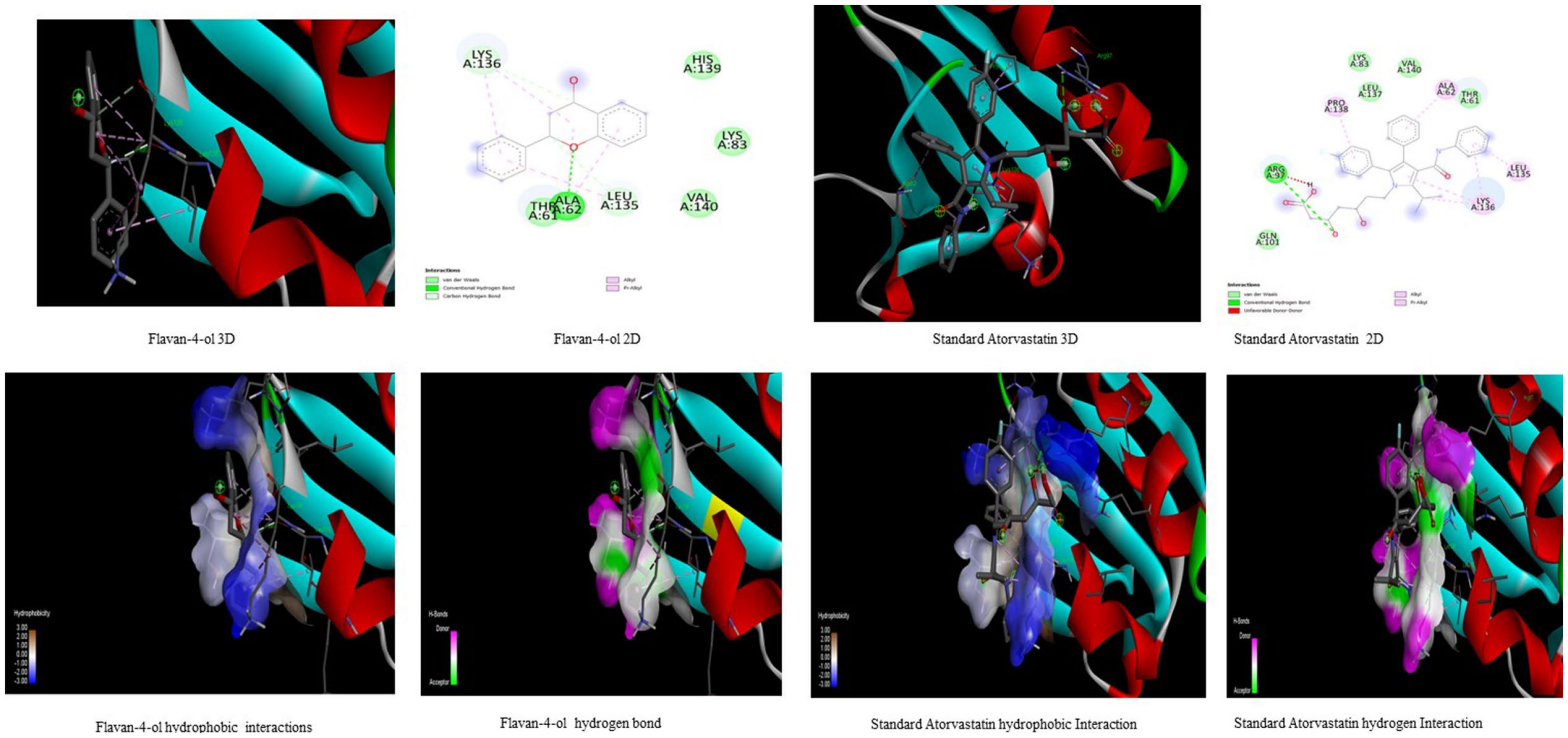
Visualization of binding interaction and hydrogen and hydrophobic bonds formed between the flavan-4-ol with Target IV Proprotein Convertase Subtilisin Kexin type 9 (PCSK9) against their positive controls.

In the *in-silico* study, stronger and more stable contact between the ligand and the target molecule is revealed by low-binding energy. The more negative the value of binding energy the more robust the complex. From the docking result, the top compounds flavan-4-ol, violaxanthin, zeaxanthin, apigenin, daidzein, luteolin, and naringenin, which are bioactive compounds, showed better binding against the positive control with the chosen targets. The flavan-4-ol is the only common molecule that docked against all four targets and established a stable complex. The binding interactions, hydrophobic interactions, and hydrogen bond formation between flavan-4-ol with the selected targets are shown in Figures 2–5.

### 3.2. Molecular docking simulation studies

Although protein–ligand docking provides effective information, it only covers the static depiction of the binding conformations of the ligand in the active region of the receptor. Thus, the integration of Newton’s equations of motion in the form of molecular dynamics provides an insight into atomic dynamics in the system throughout the defined timeline. For better understanding, an MD simulation of 100 ns was performed on all four complexes. MD trajectories analysis was used to determine the stability and fluctuation patterns of these complexes by using RMSD, RMSF (Root Mean Square Fluctuation), MM/PBSA, Radius of Gyration, and SASA (Solvent Accessible Surface Area) of the receptor atoms (Table 4).

**Table 4:** Parameters for MD analysis.

S. No	Protein–ligand Complex	Average RMSD (nm)	Average RMSF (nm)	SASA (nm\S2\N)	Potential Energy (KJ mol^−1^)
1	PCSK9-Flavan-4-ol	0.48	0.0860	61.622	−296419.2922
2	DPP4- Flavan-4-ol	0.22	0.0918	333.168	−1712766.206
3	SGLT2-Flavan-4-ol	0.36	0.1635	245.842	−998130.8998
4	ACE2-Flavan-4-ol	0.93	1.4751	259.702	−2059289.941

#### 3.2.1. Root means square deviation and root mean square fluctuation

**Figure 6:**
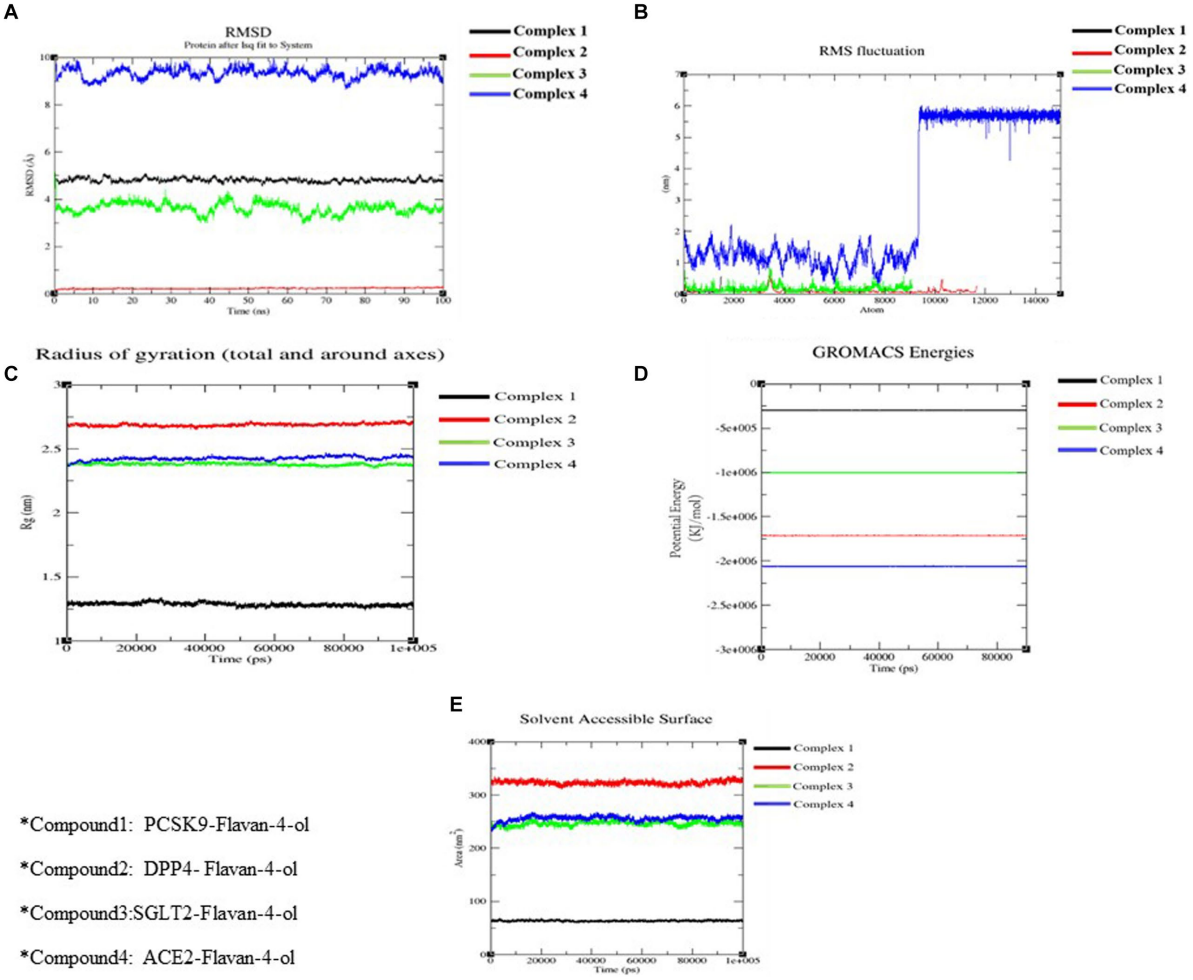
Molecular docking simulation analysis. **(A)** RMSD of all the four complexes. **(B)** RMSF of all the four complexes. **(C)** The radius of gyration of all the four complexes. **(D)** The potential energy of all the four complexes. **(E)** SASA of all the four complexes.

RMSD aids in analyzing the change in the protein structure during simulations while RMSF measures the differences in the structural confirmation of the atoms. RMSD is a crucial measure for analyzing the equilibration in the stability of complex systems during the simulation process. To measure the structural conformational differences, the RMSD of the protein backbone atoms was plotted against time. During the simulation, minor fluctuations were observed for all the complexes; however, complex 2: DPPIV-Flavan-4-ol complex had minimum RMSD as shown in Figure 6A.

RMSF is another vital parameter to consider while simulating the stability and flexibility of complex systems. The RMSF was used to determine how amino acid residues of a target protein changed their behavior upon binding to a ligand. The RMSF values for the protein’s C-alpha atoms were computed and showed against the residues. In all complexes, the amino acid residues exhibited very little variation during the simulation apart from compound 4: HACE2-flavan-4-ol complex which showed huge fluctuations and can be graphically viewed in Figure 6B.

#### 3.2.2. Radiation of gyration

Furthermore, the complex system radius of gyration (RGY) was also computed. RGY measures the root mean square distance between the protein’s atoms and the rotation axis. Being one of the critical parameters which designate the overall change in the compactness and dimensions of the protein structure throughout the simulation, elevated RGY values indicate that the protein is less compact and flexible, whereas low values indicate that the protein is very compact and inflexible. RGY values of atoms of protein backbone were plotted against time to examine the changes in structural compactness. Protein and protein–ligand complexes showed a gradual decrease in the RGY value throughout the simulation, which revealed that the test molecules induced no major structural changes in the protein (Figure 6C). Energy parameters were of great help while studying the overall stability patterns of the protein in the system. After studying the change of potential energy patterns, it was observed that there were least fluctuations for all the four complexes. The potential energies for all the complexes are shown in Figure 6D.

#### 3.2.3. Solvent accessible surface area

Solvent-accessible surface area (SASA) measures how much of a molecule’s area is available to the solvent. It is used to measure the steric availability of an atom. SASA is a significant parameter for examining the degree of receptor exposure to the surrounding solvent molecules during simulation. In general, ligand binding may cause structural changes in the receptor, causing the region in contact with the solvent to alter. SASA values of protein were plotted against time to estimate the changes in surface area. SASA for all four complexes is provided in Table 4 and depicted in Figure 6E.

#### 3.2.4. MM/PBSA – binding free energy analysis

MM/PBSAs are probably highly popular methods for predicting binding free energy because of their superior accuracy compared to most molecular docking scoring functions and lower processing requirements compared to free energy approaches. For biomolecular research on protein folding, protein–ligand binding, protein–protein interaction, and other topics, MM/PBSA has been extensively used. All complexes’ binding free energy (ΔG bind) was determined using the MM/PBSA technique for the final 20 ns (80–100 ns) of the simulated trajectories with dt 1,000 frames. Low-negative free-binding energies indicate that the test ligands have a strong affinity for binding to the target protein. Binding energies for all the complexes are given in Table 5.

**Table 5:** Binding free-energy calculations of selected complexes using MM-PBSA.

S. No	Protein–ligand Complex	ΔG bind (kJ mol^−1^)
1	PCSK9-Flavan-4-ol	-1,903.97
2	DPP4- Flavan-4-ol	−9,473.91
3	SGLT2-Flavan-4-ol	−3,247.02
4	ACE2-Flavan-4-ol	−8,699.47

### 3.3. Pharmacokinetics studies

The SWISSADME, ProTox-II, and pkCSM are used to investigate the pharmacokinetics properties, druglike nature, and medicinal chemistry of substances such as absorption, distribution, metabolism, excretion, and toxicity (ADMET) profiling. In the present study, bioactive substances flavan-4-ol, apigenin, daidzein, luteolin, and phthalic acid were classified as class V, meaning they may be dangerous if ingested in amounts of 2,000 to 5,000 mg/kg, whereas naringenin was classified as class IV, meaning it would be harmful if ingested in amounts of 300 to 2,000 mg/kg. Violaxanthin was classified as class III, meaning it is harmful if consumed at 50 < LD_50_ ≤ 300 mg/kg. Zeaxanthin class II has a lethal dose of 5 < LD_50_ ≤ 50 mg/kg as shown in Table 6. Also, the compounds were found to be neither hepatotoxic nor carcinogenic.

**Table 6:** Prediction LD_50_ value, prediction toxicity class, and pkcsM toxicity of top compounds.

Sr. No.	Compound	Prediction LD_50_ (mg/kg)	Prediction toxicity class	Ames toxicity	Max. tolerate dose human (log mg/kg/day)	Acute oral rat Toxicity (mol/kg)	Chronic oral rat toxicity (Log mg/kg_BW/day)	Hepatotoxicity
1	Flavan-4-ol	2,500	5	No	0.194	2.206	1.761	No
2	Apigenin	2,500	5	No	0.328	2.45	2.298	No
3	Daidzein	2,430	5	No	0.187	2.164	1.187	No
4	Naringenin	2000	4	No	−0.176	1.791	1.944	No
5	Violaxanthin	55	3	No	−0.384	2.132	2.054	No
6	Zeaxanthin	10	2	No	−1.058	3.496	2.603	No
7	Luteolin	3,919	5	No	0.499	2.471	2.612	No
8	Phthalic acid	2,530	5	No	0.582	1.449	2.165	No
9	Standard Atorvastatin	5,000	5	No	0.193	2.877	4.839	No
10	Standard Empagliflozin	3,000	5	No	0.25	2.554	3.51	No
11	Standard Ramipril	10,000	6	No	0.163	2.108	2.046	No

The body’s ability to absorb bioactive compounds is further influenced by their solubility and stability because of the severe pH values in the stomach and metabolism by gut microbes. A popular molecular descriptor in the investigation of drug transport characteristics, such as intestinal absorption, is Topological Polar Surface Area (TPSA). The TPSA value <140 Å shows good intestinal absorption and the TPSA value <70 Å shows good brain penetration. In the current study, the bioactive compounds luteolin, naringenin, daidzein, and apigenin have values <70 Å which means that they may act as good brain penetration compounds (Table 7).

**Table 7:** ADME (Absorption, Distribution, Metabolism, and Excretion) Prediction, and physiochemical properties of compounds.

Sr. No.	Compound	HA	HBD	HBA	MR	iLOGP	TPSA (Å)	Log S	Lipinski rule
1	Flavan-4-ol	17	1	2	66.24	2.35	29.46	−3.40	Yes
2	Apigenin	20	5	3	73.99	1.89	90.90	−3.94	Yes
3	Daidzein	19	2	4	71.97	1.77	70.67	−3.53	Yes
4	Naringenin	20	3	5	71.57	1.75	86.99	−3.49	Yes
5	Violaxanthin	4	2	4	185.80	7.22	65.52	−9.05	No
6	Zeaxanthin	42	2	2	186.76	7.23	40.46	−9.58	No
7	Luteolin	21	4	6	76.01	1.86	111.13	−3.71	Yes
8	Phthalic acid	12	2	4	40.36	0.60	74.60	−1.57	Yes
9	St. Atorvastatin	41	4	6	158.26	3.81	111.79	−5.99	Yes
10	St. Empagliflozin	31	4	7	113.41	3.40	108.61	−3.80	Yes
11	St. Ramipril	30	2	6	116.96	3.17	95.94	−2.75	Yes

**HA, No. of a heavy atom; HBD, No. of hydrogen bond donor; HBA, No. of hydrogen bond acceptor; MR, Molar refractiveness; TPSA, Topological polar surface area; iLOGP, lipophilicity; Log S, Water solubility; Lipinski rule, Drug likeness factor; St., standard.

The ability of bioactive compounds to be consumed in the human system is predicted by Lipinski’s rule of five, which is one of the most often-used characteristics of bioactive constituents that resemble pharmacological properties. The characteristics of Lipinski were determined using the canonical SMILES that were taken from the PubChem database. The flavan-4-ol, daidzein, naringenin, quercetin, luteolin, and phthalic acid showed drug-likeness properties as they follow the Lipinski rule. Flavan-4-ol has a maximum tolerated dose of 0.194 log mg/kg/day for human consumption and an acute oral rat toxicity dose of 2.206 mol/kg (Table 6).

### 3.4. In-depth analysis of identified bioactive compound – Flavan-4-ol as a potential candidate with inhibitory capacity against lifestyle diseases

**Figure 7:**
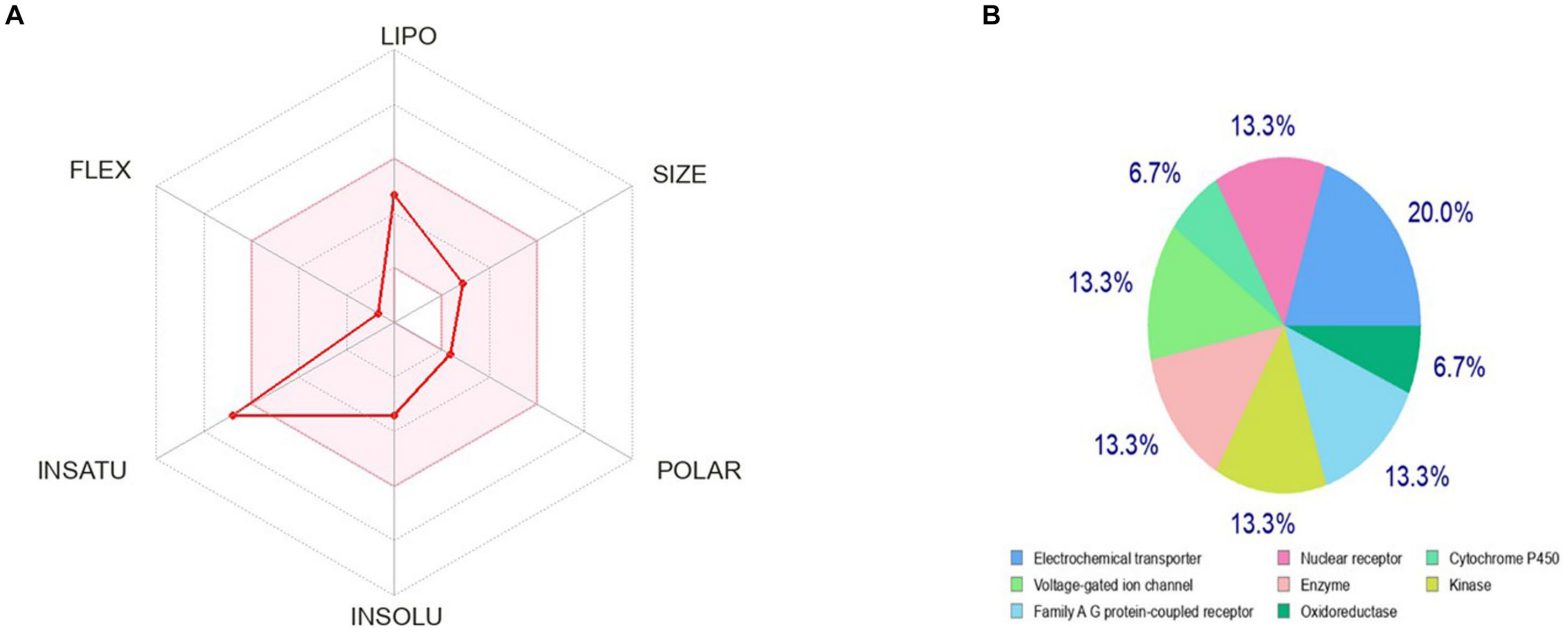
Pharmacokinetic properties and *in-silico* ADME modeling of Flavan-4-ol. **(A)** Oral bioavailability graph from the SwissADME database; the pink zone is the physicochemical space for oral bioavailability, and the red line defines oral bioavailability properties. **(B)** Target prediction.

The bioavailability radar as shown in Figure 7 revealed that the colored zone, which considered features like flexibility, lipophilicity, saturation, size, polarity, and solubility, is the ideal physicochemical region for oral bioavailability (23). According to its physical characteristics, flavan-4-ol has a molecular formula of 226.27 g/mol. There are 17 heavy total atoms, with 12 aromatic heavy atoms. In the sp3 hybridization, 0.2 carbon atoms were present. There were one rotatable bond, two hydrogen bond acceptors, and one hydrogen bond donor. The topological polar surface area was determined to be 29.46 A° and the molar refractivity to be 66.24.

The log Po/w (log P) is 2.35, the log Po/w (Xlog P3) is 2.7, the log Po/w (Wlog P) is 2.6, the log Po/w (MlogP) is 2.54, the log Po/w (SILICOS-IT) is 2.98, and the consensus log Po/w is 2.63. Overall, log *p*-values indicate that the chemical has good lipophilic characteristics. A log S (ESOL) value of −3.4, which indicates that the chemical belongs to the moderately water-soluble class, was used to analyze the substance’s water solubility.

The drug-likeness parameter is high as it is following Lipinski, Verber, and Egan rules with a bioavailability score of zero. Swiss ADME Synthetic Accessibility (SA) Score is based primarily on the assumption that the frequency of molecular fragments in ‘really’ obtainable molecules correlates with the ease of synthesis. The fragmental contribution to SA should be favorable for frequent chemical moieties and unfavorable for rare moieties. The synthetic accessibility score was found to be 3.07 which means it would not be tough to synthesize the molecule. There is no alert for PAINS, indicating the compound is quite specific in nature.

#### 3.4.1. Pharmacokinetics properties

##### 3.4.1.1. Absorption

The pharmacokinetic features of flavan-4-ol were investigated which showed Blood Brain Barrier (BBB+) with a computed probability value of 0.942; flavan-4-ol’s permeability to the BBB is 0.559 log BB and its permeability to the central nervous system is −1.676 log PS, both of which indicate a low likelihood of CNS adverse effects. According to its P-glycoprotein I inhibitor and P-glycoprotein II inhibitor scores of 0.79 and 0.88, flavan-4-ol has a low probability of being a Pgp inhibitor. As a result, it is thought to be free of serious medication interactions. A score of one indicates an inhibitor, while a score of zero indicates a non-inhibitor. This output number displays the likelihood that it is a Pgp inhibitor. Its score of 0.60 indicates that it has the lowest likelihood of being a Pgp substrate. A Pgp substrate receives a score of one, while a non-substrate receives a score of zero. According to its score of 1.00, flavan-4-ol is projected to have a low intestine absorption rate in humans (Table 8).

**Table 8:** Physicochemical and Pharmacokinetics Analysis of Flavan-4-ol.

(A) Physicochemical Properties of Flavan-4-ol (Swiss ADME)	
MW	226.27 g/mol
Heavy atoms	17
Aromatic heavy atoms	12
Fraction Csp3	0.2
Rotatable bonds	1
H-bond acceptors	2
H-bond donors	1
MR	66.24
TPSA	29.46 Å^2^
iLOGP	2.35
XLOGP3	2.7
WLOGP	2.6
MLOGP	2.54
Silicos-IT Log P	2.98
Consensus Log P	2.63
ESOL Log S	−3.4
GI absorption	High
Lipinski violations	0
Ghose violations	0
Veber violations	0
Egan violations	0
Muegge violations	0.55
Bioavailability Score	0
PAINS alerts	0
Synthetic Accessibility	3.07

(B) ADMET Features of Flavan-4-ol (pkCSM open-source tool)	
Absorption	
Intestinal absorption (human)	94.242
P-glycoprotein I inhibitor	No
P-glycoprotein II inhibitor	No
Distribution	
VDss (human)	0.478 (log L/kg)
Fraction unbound (human)	0.079 (fu)
BBB permeability	0.559 (log BB)
CNS permeability	−1.676 (log PS)
Metabolism		
CYP2D6 substrate	No
CYP1A2 inhibitor	Yes
CYP2C19 inhibitor	Yes
CYP2C9 inhibitor	No
CYP2D6 inhibitor	No
CYP3A4 inhibitor	No
Excretion	
Total Clearance	0.176 (log ml/min/kg)
Renal OCT2 substrate	No
Toxicity	
AMES toxicity	No
Max. tolerated dose (human)	0.194 (log mg/kg/day)
hERG I inhibitor	No
hERG I inhibitor	No
Oral Rat Acute Toxicity (LD50)	2.206 (mol/kg)
Oral Rat Chronic Toxicity (LOAEL)	1.761 (log mg/kg_bw/day)
Hepatotoxicity	No
Skin Sensitisation	No

(C) ADMET features of Flavan-4-ol (admetSAR open-source tool)		Probability
Absorption		
Blood–Brain Barrier	BBB+	0.9424
Human Intestinal Absorption	HIA+	1.0000
Caco-2 Permeability	Caco2+	0.7438
P-glycoprotein Substrate	Non-substrate	0.6080
P-glycoprotein Inhibitor	Non-inhibitor	0.7945
	Non-inhibitor	0.8804
Aqueous solubility		−3.2626 LogS
Caco-2 Permeability		1.3393 Log Papp, cm/s
Distribution		
Subcellular localization	Mitochondria	0.6187
Metabolism		
CYP450 2C9 Substrate	Non-substrate	0.7794
CYP450 2D6 Substrate	Non-substrate	0.8637
CYP450 3A4 Substrate	Non-substrate	0.6939
CYP450 1A2 Inhibitor	Inhibitor	0.7971
CYP450 2C9 Inhibitor	Non-inhibitor	0.6064
CYP450 2D6 Inhibitor	Non-inhibitor	0.8971
CYP450 2C19 Inhibitor	Inhibitor	0.8289
CYP450 3A4 Inhibitor	Non-inhibitor	0.9369
CYP Inhibitory Promiscuity	Low CYP Inhibitory Promiscuity	0.6713
Excretion		
Renal Organic Cation Transporter	Non-inhibitor	0.8973
Toxicity		
Human Ether-a-go-go-Related Gene Inhibition	Weak inhibitor	0.8975
	Non-inhibitor	0.8841
AMES Toxicity	Non-AMES toxic	0.6569
Carcinogens	Non-carcinogens	0.9060
Biodegradation	Not readily biodegradable	0.7280
Acute Oral Toxicity	III	0.5338
Carcinogenicity (Three-class)	Non-required	0.4707
Rat Acute Toxicity	2.6087	LD50, mol/kg

##### 3.4.1.2. Distribution

To analyze the distribution, the unbound fraction in plasma (Fu), the volume of distribution (VD), and the blood–brain barrier (BBB) permeability were taken into consideration (Tables 8B,C). The computed volume of distribution (VD) was 0.478 L/kg. The range between 0.04 to 20 L/kg is ideal for VD (38). The score of 0.559 indicates that the flavan-4-ol has a greater probability of blood–brain barrier penetration. The output value of 0.9424 is the likelihood of successfully crossing BBB. Calculations revealed that the plasma’s unbound fraction (Fu) was 0.079. This suggests that more unbound plasma fractions available for pharmacological activity.

##### 3.4.1.3. Metabolism

From the perspective of drug plasma concentration, this parameter is crucial. The database categorizes ligands as either category 0 (non-inhibitor) or category 1 (inhibitor), depending on whether they are likely to inhibit the enzyme or not. Similarly, to this, a score of one or zero represents the likelihood of being an enzyme substrate. The molecule is classified as a category 1 substrate while a category 0 non-substrate of the enzyme is indicated by the molecule (38). Due to the flavan-4-ol’s assigned score of 0.7971, it is most likely not an inhibitor of CYP1A2. The likelihood of CYP2C19 inhibition is 0.82 although the likelihood of being a CYP2C19 substrate is extremely low. Additionally, there is no evidence of CYP2D6 substrate or inhibitor. The flavan-4-ol is considered to be neither CYP3A4 substrates nor inhibitors of CYP3A4 (Tables 8B,C).

##### 3.4.1.4. Excretion

Flavan-4-ol has a low clearance rate of 0.176 mL/min/kg, which is calculated as the clearance (CL) (Table 8B). A drug’s score will be >15 mL/min/kg if its clearance rate is high, 5–15 mL/min/kg if it is moderate, and 5 mL/min/kg if it has a low clearance rate (38). Renal organic cation transporters (ROCTs) are facilitated diffusion transporters that facilitate the vectorial transport of numerous physiological chemicals and xenobiotics or drugs in the kidney, liver, and placenta cells of mammals, assisting in their absorption and elimination. There was no inhibition of Renal Oct 2 by flavan-4-ol and it did not act as a substrate for Renal OCT2 (Table 8C).

##### 3.4.1.5. Toxicity

The toxicity parameters include skin sensitization, hERG inhibition, human hepatotoxicity, AMES toxicity, carcinogenicity, rat oral acute toxicity, and hepatotoxicity. Flavan-4-ol was used in the ADMET test as a non-AMES substance and was not considered carcinogenic. There is a chance that flavan-4-ol will not cause skin sensitivity. The chemical is safe from negative reactions and has a decreased risk of carcinogenicity (0.09). A potassium ion channel that participates in the heart’s normal repolarization activity is encoded by the human ether-à-go-go-related gene (hERG) (Table 8C). Long-term QT syndrome, which can result in arrhythmia and ultimately result in mortality, can be brought on by a drug’s induction of hERG function blockage (39). With a predicted probability value of 0.89 for hERG inhibition (predictor I) and 0.88 for hERG inhibition (predictor II) for flavan-4-ol, they served as weak inhibitors and non-inhibitors of flavan-4-ol, respectively. The chance of the flavan-4-ol not easily degrading is 0.7280. All the pharmacokinetic parameters (absorption, distribution, metabolism, and excretion) are summarized in Table 8.

### 3.5. Target prediction

The observed phenotypic effects are the result of the activity of bioactive small molecules, such as metabolites, being modulated by their binding to proteins or other macro-molecular targets. It is crucial to map their targets to understand the molecular processes behind the bioactivity of bioactive small compounds and foretell any adverse effects or cross-reactivity. We can computationally find new targets for uncharacterized compounds or secondary targets for recognized molecules. Swiss Target Prediction is a web service that uses a collection of 2D and 3D similarity measurements with known ligands to precisely predict the targets of bioactive compounds. Five distinct organisms can be used to perform predictions, and mapping assumptions by homology within and across species is possible for near paralogs and orthologs (4). The flavan-4-ol outcome of the closely related receptors was calculated using the UniProt ID, ChEMBL-ID, target class, likelihood, and known actives in 2D/3D. The results were 20% electrochemical transporter, 13.3% nuclear receptor, family A-G protein receptor, kinase, enzymes, voltage-gated ion channels, and 6.7% cytochrome P450. Flavan-4-ol forecasts these other proteins as a target as well, as shown in Figure 7.

## 4. Discussion and conclusion

Millets have shown positive health impacts, including antioxidant activity, anti-diabetic, anti-tumorigenic, anti-atherogenic, and antibacterial properties (27). Regular eating of whole grain millets and their products can reduce the risk of type II diabetes, gastrointestinal malignancies, cardiovascular disease, and a variety of other ailments (41). Most millets have a carbohydrate content between 60 and 70%, with the majority being non-starchy polysaccharides, contributing to millets’ many health advantages (42). According to various epidemiological studies, eating millet enhances the immune system, detoxifies the body, lowers the risk of cancer, boosts energy, improves brain and muscular systems, and raises immunity in the respiratory system (3, 43). Typically, millets are eaten with the seed coat, which is high in phenolics, dietary fiber, minerals, and vitamins and is more beneficial to human health than other whole-grain cereals (26, 43).

The computational approaches investigated novel compounds from millet sources in terms of their interactions with DPPIV, SGLT-2, hACE, and PCSK9. A structure-based virtual screening method called molecular docking discovers active inhibitors based on predictions of the binding affinities and molecular interactions between ligand molecules (or inhibitors) and their corresponding target proteins or enzymes. The binding affinity of active inhibitors is typically evaluated using a flexible docking simulation methodology. For the protein–ligand complex created with low energy conformation, the most advantageous binding mechanism docking poses is examined (37). By docking, the optimal binding orientation of ligands for their corresponding target molecules is discovered.

DPPIV is an integral membrane aminopeptidase and member of the prolyl oligopeptidase that was initially identified as a T-cell differentiation antigen (CD26) and was reported on the diverse groups of epithelial cells, viz., kidney, liver intestine, prostate, lung, and placenta. DPPIV is a major glycemic mediator used to control type 2 diabetes mellitus which is associated with severe life-threatening coronary diseases such as stroke, heart failure, and many more cardiovascular adverse effects (8, 15). Inhibition of DPPIV by bioactive compounds provides proof as a tool for the treatment of type 2 diabetes mellitus (8). DPPIV inhibition reduces inflammation and immune system activation, which are frequent characteristics of diabetes and hypertension, indicating that these processes may play a significant part in DPPIV-mediated kidney damage. Sitagliptin and vildagliptin, two DPPIV inhibitors that are now available on the market, exhibit notable hypoglycemia effects. However, they can also cause rashes, upper respiratory tract infections, and hypersensitivity responses (44–46). Therefore, a trustworthy and tried method for finding new hypoglycemia medications is the discovery of DPP-IV inhibitors with novel structures, particularly among the secondary metabolites of plants. The results of our docking analysis indicate that among 50 bioactive compounds docked against DPPIV, apigenin, zeaxanthin, flavan-4-ol, and violaxanthin had better binding interactions when compared to the standard empagliflozin. Furthermore, the number of hydrogen bonds formed between the target and inhibitor was found to be a significant predictor of stable complex formation. For instance, apigenin formed four hydrogen bonds with the target protein while the other compounds formed one hydrogen bond. Our findings suggest that apigenin, zeaxanthin, flavan-4-ol, and violaxanthin hold promise as potential candidates for further study in drug discovery against DPPIV.

Type-2 diabetes is a metabolic disorder characterized by high levels of glucose in the blood. Renal glucose reabsorption is an important factor in maintaining elevated blood glucose levels. Sodium-glucose cotransporters, particularly SGLT-2, play a significant role in glucose reabsorption from the kidneys (25). SGLT-2 inhibitors have emerged as a new class of antihyperglycemic agents that help manage type-2 diabetes by inhibiting the SGLT-2 pathway of glucose reabsorption in the kidneys, leading to increased urinary excretion of excess glucose and lowering blood sugar levels (47). This mode of action is insulin-independent, which means that these inhibitors can be used alone or in combination with other antidiabetic agents to improve glycemic control. Furthermore, SGLT-2 inhibitors have been found to exert nephroprotective effects in patients with chronic kidney disease. As type-2 diabetes is a major health concern worldwide, the role of SGLT-2 inhibitors in managing this chronic condition cannot be overstated (47). The results of docking bioactive compounds against the macromolecule SGLT-2 showed that flavan-4-ol, daidzein, luteolin, and naringenin had more negative-binding energies than the standard Empagliflozin. This indicates that the tested compounds showed greater stability in complex with SGLT-2. Flavan-4-ol demonstrated the highest stability, forming two hydrogen bonds with seven different amino acids in SGLT-2. Daidzein, luteolin, and naringenin also showed stability and formed three hydrogen bonds with amino acids in SGLT-2. These findings, obtained via a structure-based drug-design method, are critical in the development of drugs that can effectively target SGLT-2. Moreover, SGLT-2 inhibitors have been found to be a promising new type of anti-diabetic drug. The use of SGLT-2 inhibitors has been proven to be effective in reducing blood glucose and weight without increasing the risk of hypoglycemia. In addition, a meta-analysis demonstrated that SGLT-2 use has significant cardiovascular and renal protective effects.

Human angiotensin-converting enzyme (hACE) control leads to the management of hypertension which poses a serious risk of developing coronary disease, heart failure, stroke, and a variety of other cardiovascular diseases (48). It plays an integral role in the control of blood pressure through the integration of the Angiotensin 2 pathway synthesis. A high concentration of Angiotensin 2 affects the renal tubule to retain sodium and water which further results in hypertension (48). The maintenance of cardiovascular homeostasis depends on the renin-angiotensin system. ACE inhibition or angiotensin II receptor blockade is the mainstay of therapy for several cardiovascular disorders. Angiotensin-(1-7) levels in plasma and tissues may rise as a result of hACE inhibition, which prevents the conversion of angiotensin-(1-7) to angiotensin (49). Current clinical uses for hACE inhibitors include the management of hypertension, endothelial dysfunction, congestive heart failure, myocardial infarction, and renal illness (including diabetic nephropathy) (23). Our docking analysis identified five bioactive compounds as potent hACE inhibitors, with compounds violaxanthin, zeaxanthin, flavan-4-ol, and daidzein being the most effective. These compounds formed hydrogen bonds with key active site residues and exhibited higher binding energies and Ki values than Ramipril. Further *in vitro* and *in vivo* studies are warranted to confirm the efficacy of these compounds as potential therapeutic agents for hypertension and related cardiovascular diseases.

A serine protease called PCSK9 plays an integral role in the regulation of the cholesterol level of the body. It binds to hepatic-specific LDL (low-density lipoprotein) receptors and increases the intracellular degradation of the intricate LDL receptor, hence decreasing blood LDL clearance. Despite being synthesized to a lesser amount in other organs, PCSK9 is primarily released by the liver. In addition to its well-known role in the hepatic LDL receptor-mediated pathway, PCSK9 has also been linked to the claim that it may prevent vascular inflammation during atherogenesis (23). When LDL receptors are blocked, there is a rise in LDL concentration, which increases the risk of developing cardiovascular disease and stroke. The gain-of-function mutation of PCSK9 results in autosomal-dominated familial hypercholesteremia. Inhibition of PCSK9 is considerable promise for the management of hypercholesterolemia and its associated cardiovascular disease. The docking study identified phthalic acid, naringenin, daidzein, and flavan-4-ol as PCSK9 inhibitors with varying binding energies, the number of hydrogen bonds formed, and inhibition constants. The findings provide valuable insights for the design of more potent PCSK9 inhibitors for the treatment of hypercholesterolemia.

All the top four substances exhibit higher binding affinities than the standard. The binding energy was found through an *in-silico* analysis to indicate a stronger and more stable connection between the ligand and the target molecule. Bioactive substances including flavan-4-ol, violaxanthin, zeaxanthin, apigenin, daidzein, luteolin, and naringenin have stronger binding energies with the targeted molecules than the more often used atorvastatin, empagliflozin, and ramipril. The stronger the complex, the higher the negative binding energy value. Only the flavan-4-ol is the most prevalent molecule that docked against all four targets, and it formed a stable complex with all four targets.

Pharmacokinetics analysis supported the MD data. Also, the 100 ns MDs verified the examined compounds’ affinity by demonstrating improved stability in the receptor-binding region. MM/PBSA binding Free Energy Analysis depicted low negative free binding energies indicating that the test ligands had a strong affinity for binding to the target protein (34). Among the four complexes, the PCSK9-flavan-4-ol had low binding energy −1,903.97 ΔG bind (kJ mol^−1^) as shown in Table 5. They were then put through an MD simulation trajectory. The results of the RMSD analysis showed that the DPPIV- flavan-4-ol complex had minimum, and RMSF complex 4: HACE2-flavan-4-ol showed huge fluctuations; the analysis proceeds further for RGY and SASA during the whole 100 ns MD trajectory. Overall, the four complexes show fluctuations for more stability. The root means the square distance between a protein’s atoms and its rotational axis is measured by RGY. It is one of the crucial variables that describe the overall change in the compactness and dimensions of the protein structure during the simulation. Low RGY values imply a protein that is extremely compact and inflexible, whereas elevated values denote a protein that is less compact and flexible. Protein’s backbone RGY values were plotted over time to observe how the compactness of the structure changed over time. Throughout the simulation, the RGY value of the protein and protein–ligand complexes gradually decreased, indicating that the test compounds did not significantly alter the protein’s structural composition (Figure 6).

The LD_50_ can measure acute toxicity, and the six classes of toxicity classifications are outlined by the Globally Harmonized System of Classification and Labelling of Chemicals (GHS) (50). In this study, flavan-4-ol, apigenin, daidzein, luteolin, and phthalic acid were classified as class V, meaning they may be dangerous if ingested in amounts of 2000 to 5,000 mg/kg, and naringenin was classified as class IV, meaning it would be harmful if ingested in amounts of 300 to 2000 mg/kg.

Bioactive compounds, such as violaxanthin and zeaxanthin, have been found to be neither hepatotoxic nor carcinogenic. They are more permeable than other compounds due to their solubility, stability, and metabolism by gut microbes. The TPSA value of luteolin, naringenin, daidzein, and apigenin has been used to measure their capacity to be orally active in the human system. Lipinski’s rule of five has been used to predict the drug-likeness properties of bioactive compounds, except for zeaxanthin.

Bioactive compounds are important for discovering new drugs, but animal models are not reliable predictors of human toxicity. We found that the compound flavan-4-ol is best docked to all four targets of lifestyle diseases, and MD simulation analysis further strengthens our finding that the flavan-4-ol forms a better stability complex with all the targets. ADMET profiles substantiate the candidature of the flavan-4-ol bioactive compound to be considered for trial as an inhibitor of targets DPPIV, SGLT2, PCSK9, and hACE. We suggest that more research is conducted, taking Flavon-4-ol into account when producing new medicines from millets. Multi-target therapeutic candidates can be created from it to suppress the biochemical pathway of diseases diabetes, hypertension, and atherosclerosis.

## Data availability statement

The original contributions presented in the study are included in the article/supplementary material, further inquiries can be directed to the corresponding author.

## Author contributions

KN and NS: conception, design, methodology, statistical analysis, and writing manuscript. GT and MI: simulation analysis and manuscript writing. TN: visualization and review. RB, AM, and CG: writing—review and editing. All authors contributed to the article and approved the submitted version.

## Conflict of interest

The authors declare that the research was conducted in the absence of any commercial or financial relationships that could be construed as a potential conflict of interest.

## Publisher’s note

All claims expressed in this article are solely those of the authors and do not necessarily represent those of their affiliated organizations, or those of the publisher, the editors and the reviewers. Any product that may be evaluated in this article, or claim that may be made by its manufacturer, is not guaranteed or endorsed by the publisher.
